# Interactions between inflammatory mediators and corticosteroids regulate transcription of genes within the Kynurenine Pathway in the mouse hippocampus

**DOI:** 10.1186/s12974-016-0563-1

**Published:** 2016-05-03

**Authors:** Alexandra K. Brooks, Marcus A. Lawson, Robin A. Smith, Tiffany M. Janda, Keith W. Kelley, Robert H. McCusker

**Affiliations:** Neuroscience Program, University of Illinois at Urbana-Champaign, Urbana, IL 61801 USA; Department of Animal Sciences, University of Illinois at Urbana-Champaign, Urbana, IL 61801 USA; Integrative Immunology and Behavior Program, University of Illinois at Urbana-Champaign, Urbana, IL 61801 USA; Department of Pathology, University of Illinois at Urbana-Champaign, Urbana, IL 61801 USA; 250A, Edward R. Madigan Laboratory, 1201W. Gregory Dr., Urbana, IL 61801-3873 USA

## Abstract

**Background:**

Increased tryptophan metabolism towards the production of kynurenine via indoleamine/tryptophan-2,3-dioxygenases (DOs: Ido1, Ido2, and Tdo2) is strongly associated with the prevalence of major depressive disorder in patients and the induction of depression-like behaviors in animal models. Several studies have suggested that activation of the immune system or elevated corticosteroids drive DO expression; however, mechanisms linking cytokines, corticosteroids, and DOs to psychiatric diseases remain unclear. Various attempts have been made to correlate DO gene expression within the brain to behavior, but disparate results have been obtained. We believe that discrepancies arise as a result of the under-recognized existence of multiple mRNA transcripts for each DO. Unfortunately, there are no reports regarding how the multiple transcripts are distributed or regulated. Here, we used organotypic hippocampal slice cultures (OHSCs) to directly test the ability of inflammatory and stress mediators to differentially regulate DO transcripts.

**Methods:**

OHSCs were treated with pro-inflammatory mediators (interferon-gamma (IFNγ), lipopolysaccharide (LPS), and polyinosine-polycytidylic acid (pI:C)) with or without corticosteroids (dexamethasone (Dex: glucocorticoid receptor (GR) agonist), aldosterone (Aldo: mineralocorticoid receptor (MR) agonist), or corticosterone (Cort: GR/MR agonist)).

**Results:**

IFNγ induced Ido1-full length (FL) and Ido1-variant (v) expression, and surprisingly, Dex, Cort, and Aldo interacted with IFNγ to further elevate expression of Ido1, importantly, in a transcript dependent manner. IFNγ, LPS, and pI:C increased expression of Ido2-v1 and Ido2-v3 transcripts, whereas only IFNγ increased expression of Ido2-v2. Overall Ido2 transcripts were relatively unaffected by GR or MR activation. Naïve mouse brain expresses multiple Tdo2 transcripts. Dex and Cort induced expression of only one of the three Tdo2 transcripts (Tdo2-FL) in OHSCs.

**Conclusions:**

These results establish that multiple transcripts for all three DOs are expressed within the mouse hippocampus, under the control of distinct regulatory pathways. These data identify a previously unrecognized interaction between corticosteroid receptor activation and inflammatory signals on DO gene expression, which suggest that corticosteroids act to differentially enhance gene expression of Ido1, Ido2, and Tdo2.

## Background

The lifetime prevalence of major depressive disorder (MDD) is almost 15 % [1] with nearly 10 % of the USA population taking anti-depressants on any occasion [2]. Within the general population, prevalence is five to ten times higher in patients with a known medical illness [3], particularly when this medical illness is a chronic inflammatory condition [3]. For example, people with multiple sclerosis have a prevalence rate of MDD up to 50 % [4]. Over the past 20 years, an association between the immune system and MDD has been clearly established. Studies have shown that patients with MDD have elevated levels of immunomodulatory factors (pro-inflammatory cytokines) within the circulation and increased expression of pro-inflammatory cytokines in the central nervous system, neuroinflammation [5]. During chronic administration of the pro-inflammatory cytokine IFNα to patients with hepatitis C or malignant melanoma, up to 45 % of patients eventually exhibit elevated symptoms of MDD [6–8]. As such, patients who have undergone a chronic immune challenge express a variety of depressive symptoms [7, 9]. Now, one of the most pressing issues is to determine how inflammatory cytokine signaling is linked to a pathway responsible for depression-like behaviors [10]. The answer to this question could aid in development of new anti-depressant drugs that would benefit a large percentage of the population. This is especially pertinent considering that anti-depressants are therapeutically effective in only 15 % of patients after accounting for the placebo effect [11].

Several reports have identified polymorphisms associated with DO expression, an elevated immune response, and dysregulation of the hypothalamic-pituitary-adrenal axis. Polymorphisms in the Ido1 gene are associated with both treatment efficiency of anti-depressants [12] and symptomology of depression [13]. A polymorphism in the promoter region of the Ido1 gene (rs9657182, CC genotype) is a risk factor for patients to develop depression after immunotherapy [13]. An additional study [14] found that patients are more likely to develop symptoms of depression following immunotherapy if they harbored the “high producer” allele (IFNγ + 874, T allele) that is associated with elevated IFNγ expression [15, 16]. This allele is also associated with increased DO activity [14, 17], suggesting that this genotype may be a risk factor for increased interferon/DO-induced depression [18]. There are also functional polymorphisms in the GR gene associated with symptoms of depression [19–21]. These data provide accumulating evidence for convergence of immune (IFNγ → DO) and stress (HPA dysregulation/corticosteroid → GR activation → DO expression) pathways involved in depression. The least characterized aspect of this convergence is the interaction between these two pathways as regulatory pathways controlling DO expression.

Extensive research has illustrated mechanisms by which inflammatory challenge induces a depression-like phenotype with rodent models. Administration of lipopolysaccharide (LPS), polyinosine-polycytidylic acid (pI:C), or infection with *Mycobacterium bovis* evokes peripheral and CNS cytokine expression followed by the manifestation of depression-like behaviors such as helplessness/despair (increased immobility during tail suspension test, TST) and anhedonia (decreased sucrose preference [22, 23]. Importantly, the development of depressive symptoms is tied to elevated tryptophan metabolism to kynurenine via the DOs [28] which are rate limiting for the metabolism of tryptophan to kynurenine (Kyn) via the *Kynurenine Pathway* [26–29]. Ido1, Ido2, and Tdo2 messenger RNA (mRNA) expression are induced within the brain and peripheral tissues by LPS and bacterial infection, pI:C and viral infection, and systemic and CNS administration of pro-inflammatory cytokines [25–28, 30–34]. Inflammatory cytokines released during an immune challenge include IFNγ, IFNα, interlukin-1 beta (IL-1ß), IL-6, and tumor necrosis factor alpha (TNFα). These cytokines are necessary to activate both lymphoid and myeloid cells in the periphery and induce neuroinflammation, but their presence is not sufficient to induce depression-like behaviors. DO induction is requisite for depressive-like behaviors as genetic knockout of Ido1 gene transcription (Ido1^KO^ mice) inhibits inflammation-dependent depression-like behaviors [28, 29, 35]. Kyn produced by the DOs is not directly responsible for behavioral changes. Kyn is further metabolized by non-rate-limiting enzymes: kynureninase (Kynu) + kynurenine 3-monooxygenase (KMO) leading to quinolinic acid (QuinA) production or by kynurenine aminotransferase (Kat2) that generates kynurenine acid (KynA) [36]. QuinA and KynA directly interact with neurons to interfere with neurotransmitter activity, and they are believed to be the end products responsible for the behavioral changes that are initiated and dependent on DO expression. KynA acts on neurons to decrease glutamate and acetylcholine signaling, whereas QuinA enhances glutamate receptor activation on neurons, suggesting that Kyn metabolites play distinct roles in the pathophysiology and etiology of a variety of neurological conditions [37, 38].

Similar to the effects of cytokines, a recent study has shown that elevated corticosteroids increase Ido1 expression in the mouse hippocampus [39]. Chronic unpredictable mild stress increases both serum corticosterone and Ido1 and leads to depression-like behaviors of rats [40]. Additionally, swimming stress is well known to elevate blood corticosterone levels in mice, and when combined with systemic LPS injection, there is an induction of Ido2 expression in the mouse hippocampus [32]. These data suggest that inflammatory cytokines and stress hormones can act both alone and together to induce Ido1 and Ido2 expression. Although several papers indicate elevated Tdo2 expression in the liver following corticosteroid administration [41, 42], there are no studies investigating the regulation of Tdo2 within the brain. Unfortunately, the DO specificity, inflammatory-mediator specificity, and corticosteroid-receptor specificity or their interactions have not been defined.

**Fig. 1:**
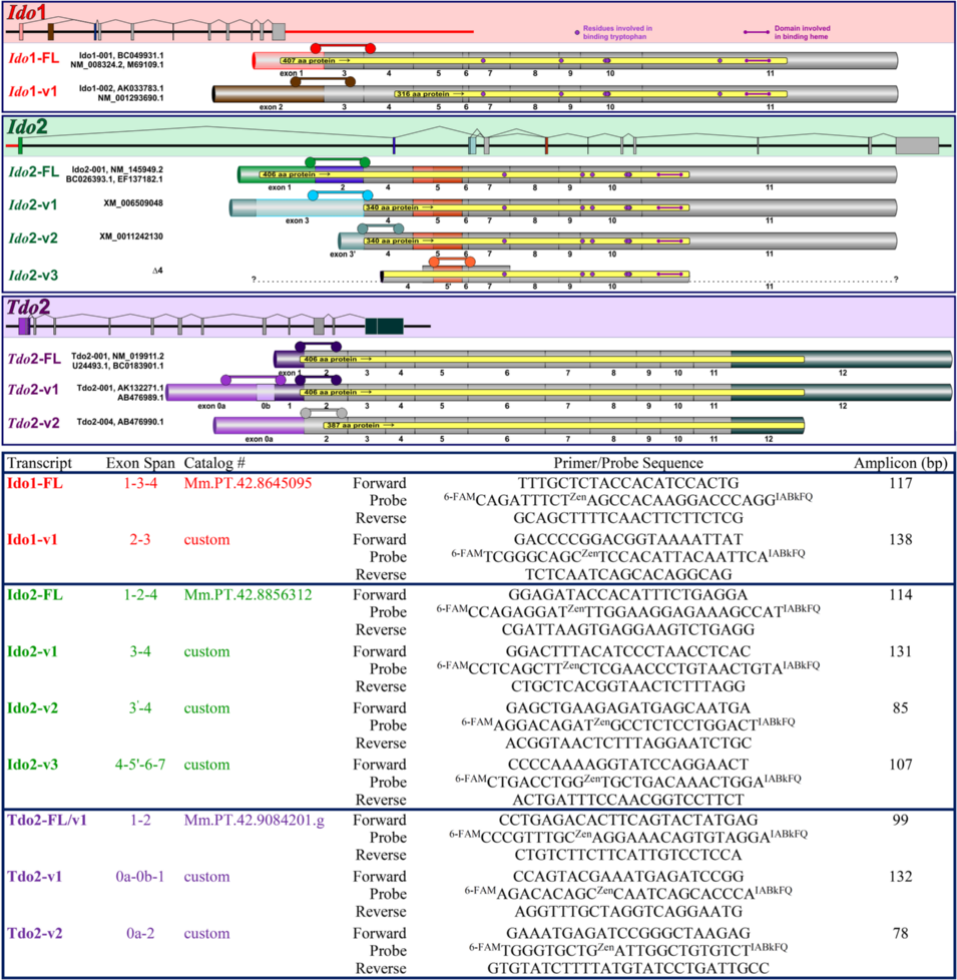
Gene, transcript, and qPCR assay information for murine Ido1, Ido2, and Tdo2. *Top*: Gene structure (to scale) is shown in the shaded areas for each of the three DOs with each gene having either 11 or 12 exons. Ido1 and Ido2 are contiguous on chromosome 8, separated by only 7,733 bp (*red bar*). Tdo2 is located on chromosome 3. Each of the three genes are transcribed into multiple transcripts (exon inclusion for unique transcripts are shown below the gene structure, approximate scale) which encode full-length (FL) and variant (v) forms of each protein (protein coding areas shown in *yellow* within each transcript). Splicing patterns were taken from two databases (Ensemble and NCBI, neither of which describe all of the variants) and two manuscripts [47, 48], all with unique naming criteria. Thus, simplified names for each transcript are used in the manuscript (shown on the left, *colored text*) and database/manuscript sequence names are provided in black text. Ido2-v3 (Δ4 described in [47]) is not listed in either database; a partial sequence of this transcript has been published (*thick bar area*), but when the transcript was overexpressed, it encodes an active enzyme [47] that lacks 12 amino acids (due to the absence of the first 36 bp of exon 5). Invariant exons that are used for all mRNA transcripts within DO are *gray*; exons that vary in usage or length are colored. *Bottom*: All qPCR assays were purchased from IDT. User-specific (custom) assays were designed using the IDT PrimerQuest® Design Tool. It is not possible to design an assay specific to Tdo2-FL as its complete sequence lies within Tdo2-v1 (however data within this manuscript indicate distinct differences in expression and regulation of Tdo2-FL and Tdo2-v1). Ido2-v3:Δ4, Tdo2-FL/-v1/-v2 transcript names, and Tdo2 exon 0a and 0b exon designations are shown in the figure to agree with published nomenclature [47]. Specifics shown for each qPCR assay include location (assay location is also shown in upper panel ), catalog numbers (if predesigned by IDT), primer/probe sequences, and confirmed amplicon size

Our first attempts to define the interaction between inflammation and stress for the regulation of DO within the brain were met with mixed results. Results differed when we used quantitative PCR (qPCR) assays that amplified the 5′ regions of the DOs versus qPCR assays that amplified 3′ regions of the DOs. This discrepancy was addressed by purchasing or designing qPCR assays that specifically amplified the unique splice variants for each DO (Fig. 1). Since both inflammatory stimuli and stress hormones induce depression-like behaviors and DO upregulation, we hypothesized that inflammatory stimuli and glucocorticoids interact to regulate DO expression. More importantly, we hypothesized that splice variants for the DO exist as a means to differentially regulate DO expression dependent on the type of stimuli presented to the brain. We found that both Dex and Cort (but not Aldo) attenuate LPS and pI:C-induced pro-inflammatory cytokine expression, a typical anti-inflammatory response. Surprisingly, instead of diminishing the effect of inflammatory stimuli on Ido expression, corticosteroids upregulated IFNγ-induced Ido1 expression in a transcript-dependent manner. The corticosteroids had minimal effects on Ido2 expression, but Ido2 was induced by both LPS, pI:C and IFNγ, whereas Ido1 was only induced by IFNγ. Only one of three Tdo2 transcripts was induced by corticosteroids. These data uncover novel pathways that can be manipulated to control DO expression.

## Methods

### Mice

C57BL/6J founders were purchased from Jackson Laboratories. Pups were supplied from a breeding colony established and maintained in the University of Illinois’s Institute for Genomic Biology animal facility. All animal care and use procedures were conducted in accordance with the Guide for the Care and Use of Laboratory Animals (National Research Council) and approved by the Institutional Animal Care and Use Committee.

### Reagents

Hank’s balanced salt solution (HBSS, SH30030.03), heat-inactivated horse serum (SH30074.03), and MEM (SH30024.02) were purchased from Hyclone (Logan, UT). D-glucose (15023-021) was from Gibco (Carlsbad, CA). Gey’s balanced salt solution (GBSS, G9779), pI:C (tested at 10 μg/ml, P0913), Dex (tested at 12.5 μM, D4902), Aldo (tested at 100 nM, A9477), Cort (tested at 1 μM, 27840), and LPS (tested at 2 μg/ml, *Escherichia coli* 0127:B8, L-3129) were from Sigma (St. Louis, MO). Recombinant mouse IFNγ (tested at 1 μg/ml, 315-05) was from Peprotech (Minneapolis, MN). Since there are no publications describing the corticosteroid responsiveness or corticosteroid x inflammation interactions of organotypic hippocampal slice cultures (OHSCs), we used treatment doses to assure that consistent OHSC responses to both types of stimuli were present.

### Organotypic hippocampal slice cultures

OHSCs were prepared from 7- to 10-day old pups as previously described [30]. Briefly, mice were decapitated and brains were removed, followed by extraction of the hippocampi from both brain hemispheres. Transverse slices (350 μm) were prepared with a McIlwain tissue chopper (Campden Instruments Ltd, UK) then transferred onto porous (0.4 μm) transparent membrane inserts (30 mm in diameter; EMD Millipore) with one slice from each of six different mice per insert. Inserts were placed into six-well culture plates with 1.25 ml of culture medium composed of 25 % heat-inactivated horse serum, 25 % Hank’s Balanced Salt Solution (HBSS), 50 % Modified Eagle’s Medium (MEM), 1× penicillin/streptomycin (Pen/Strep), 0.5 % D-glucose, and 15 mM 4-(2-hydroxyethyl)-1-piperazineethanesulfonic acid (HEPES buffer pH 7.4). Plates were maintained in a humidified incubator (5 % CO_2_, 95 % atmospheric air) at 37 °C. Medium was changed every 2–3 days. On day 7 in culture after slices had recovered from the inflammatory response associated with slice preparation [30], OHSCs were rinsed several times and incubated for 2 h in serum-free conditions (Dulbecco’s MEM, Pen/Strep, HEPES, and 150 μg/ml bovine serum albumin) before a replacement of the serum-free medium and addition of treatments. Six hours following addition of treatments, supernatants and tissues were collected and stored at −80 °C for further analysis. A 6-h treatment duration was chosen based on our finding that the maximal cytokine and Ido1 mRNA responses to LPS occurred after this duration [30].

### Reverse transcription and real-time RT-PCR

Total RNA from OHSCs was extracted using a commercially available kit as per the supplier’s instructions (E.Z.N.A. Total RNA Kit II, Omega Bio-Tek, Norcross, Georgia). RNA was reverse transcribed to complementary DNA (cDNA) using a high-capacity cDNA reverse transcription kit (Applied Biosystems, Grand Island, NY). The cDNA samples were analyzed for steady-state mRNA levels (i.e., gene expression) by qPCR using TaqMan universal PCR master mix and the Prism 7900 thermocycler (Applied Biosystems, Foster City, CA). Data were analyzed using the comparative threshold cycle method (GAPDH expression used to normalize target gene expression [27]. Changes in target cDNA levels were analyzed by comparing 2^−ΔΔCts^, where Ct is the cycle threshold.

### DO transcripts and qPCR assay design

Much of this work was prompted by data generated while investigating the mRNA expression of DOs across mouse tissues. Results varied dependent on the qPCR assay location. However, the average mouse gene has three mRNA isoforms (seven for human) [43], suggesting that a single qPCR assay may not detect the true expression level of a gene or intimacies of its tissue/cellular distribution and regulation. To determine the relevance of gene splicing to the DOs, it is important to outline the multiple transcripts for the three DOs. The gene structures and transcripts (two for Ido1, four for Ido2, and three for Tdo2) are illustrated as are the qPCR assays used to quantify each transcript (Fig. 1 bottom). Correct amplicon size for each qPCR assay was confirmed by gel electrophoresis. Proteins transcribed from these transcripts (~51 kDa Ido1-FL [24, 44], ~45 kDa Ido1 (probably FL but possibly v1) [45, 46], ~54 kDa Ido2-FL [24, 47], <54 kDa Ido2-v3 [47], ~46 kDa Tdo2-FL [48], ~46 kDa Tdo2-v1 [48], and ~44 kDa Tdo2-v2 [48]) have been expressed and found to encode enzymatically active proteins. Ido2-v1 and Ido2-v2 transcripts are predicted to encode variant DO proteins that are distinct from the confirmed active Ido2-FL and Ido2-v3 encoded proteins (Fig. 1 top, Ensemble and NCBI databases); enzymatic activity of these predicted proteins has not been tested.

### Cytokine levels

Media collected from slice cultures were analyzed for TNFα and IL-6 levels using BD OptEIA™ ELISA kits (BD Biosciences; San Diego, CA) following the manufacturer’s instructions.

### Statistics

All data are presented as mean ± SEM. Gene expression data are means of three to four independent OHSC preparations. Two-way analysis of variance was performed using SigmaPlot 13.0 software and a 2 × 6 arrangement of treatments. Post hoc analysis for multiple comparisons was performed only in the presence of a significant interaction, as assessed by Holm-Šídák method. Significance was set at *p* ≤ 0.05 for all comparisons.

## Results

### Ido1, Ido2, and Tdo2 transcript expression differ between tissues and across brain regions

**Fig. 2:**
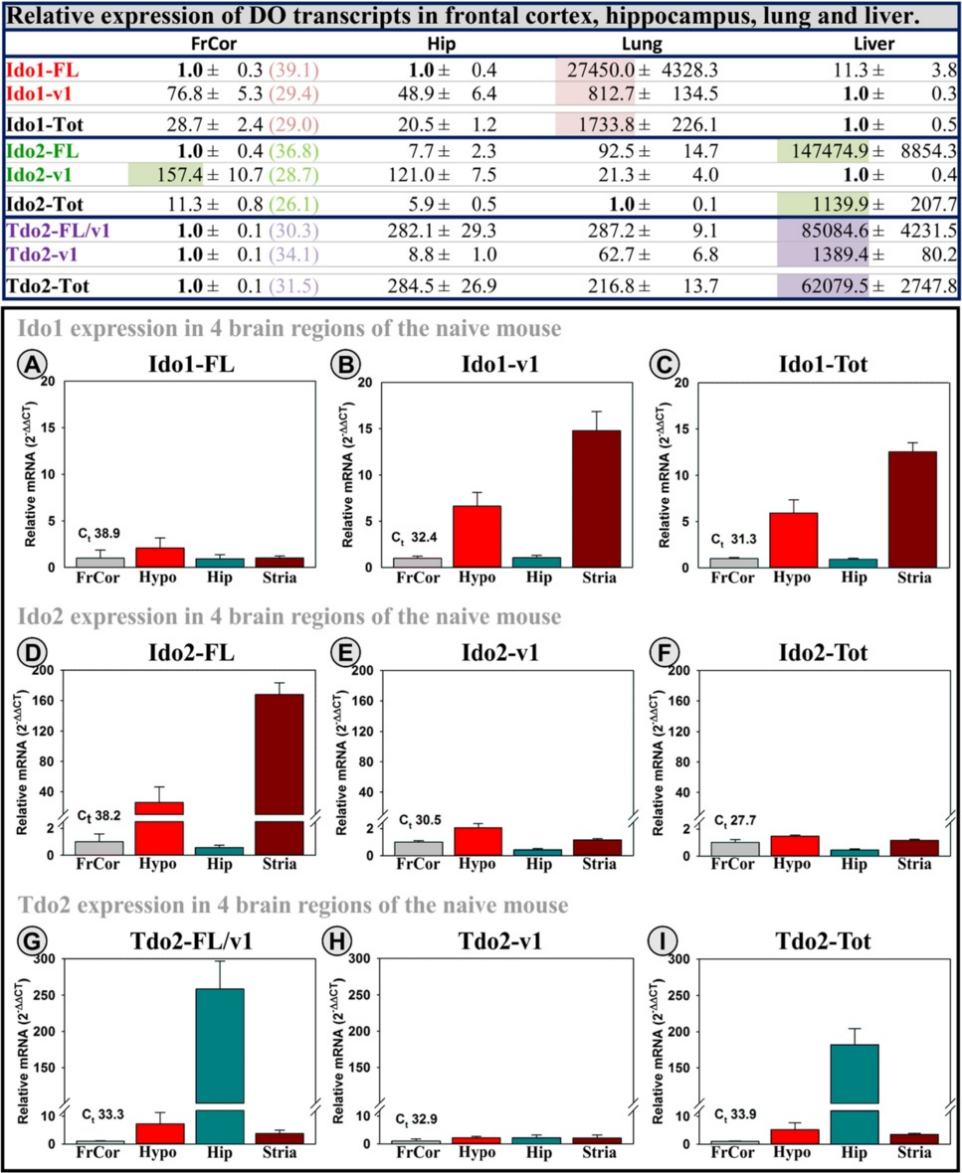
Ido1, Ido2, and Tdo2 transcript expression differ between tissues and across brain regions. *Top*: Two brain regions (frontal cortex, FrCor; hippocampus, Hip), the lung and liver, were collected from naïve mice (*n* = 6). Expression of two transcripts for each DO is presented to illustrate tissue and brain region specificity. The sample type with the lowest expression was set to 1.0, with other samples showing relative expression levels within transcript (i.e., across row, *C*_t_ values for FrCor are shown to illustrate relative amplification across transcripts). The tissue with the highest relative expression for each transcript is highlighted for emphasis. Total DO expression (Ido1-Tot, Ido2-Tot, and Tdo2-Tot) was assessed using assays that span exons conserved across all transcripts within each gene. *Bottom*: Similarly, brain regions were collected to quantify differences in expression levels across additional brain regions of naïve mice (*n* = 4). Expression within the frontal cortex was set to 1.0 for all transcripts, and other regions show expression relative to the FrCor. *C*_t_ values for each transcript (**a**: Ido1-FL, **b**: Ido1-v1, **c**: Ido1-Tot, **d**: Ido2-FL, **e**: Ido2-v1, **f**: Ido2-Tot, **g**: Tdo2-FL/v1, **h**: Tdo2-v1, I: Tdo2-Tot) within the FrCor are shown to indicate relative amplification level during the qPCR reaction (Ido1-FL is essentially absent in the naïve mouse brain; samples without a calculated *C*_t_ value are assigned a value of 40 for presentation). For both data sets, mice (10- to 12-week old) were euthanized under CO_2_ and then perfused with cold saline to minimize blood content within samples prior to tissue or brain region excision. Samples were then processed for qPCR analysis as described in the “Methods” section for OHSC samples

Although gene expression has been published for several of the DO transcripts, a quantitative comparison of full length (FL) versus variant (v) transcripts has not been performed for the three DOs within the brain or across tissue types. Comparing brain regions to the lung and liver, the Ido1-FL transcript is essentially absent (*C*_t_ values ≥37) in mouse brain (Fig. 2 top), but it is expressed in the mouse liver and abundantly so in the lung. In contrast, Ido1-v1 expression is lowest in liver but is expressed in both the frontal cortex and hippocampus (*C*_t_’s ~32) and is again highest in the lung. The Ido2-FL transcript is lowest in the mouse brain (C_t_’s ~32), expressed in the lung, but highest in the liver, essentially opposite of the expression pattern for the Ido2-v1 transcript. Unlike either Ido1 or Ido2, Tdo2 transcripts are lowest in the frontal cortex of the mouse brain (*C*_t_’s ~34), considerably higher in the hippocampus and lung, but highest in the liver. These data indicate that tissues differentially express specific transcripts for each of the DOs. Thus, quantifying DO expression within different tissue may require the use of different assays. These data also suggest that distinct mechanisms must exist to determine which transcript will be expressed. More importantly, these data indicate that a single qPCR assay does not reflect changes in DO transcript levels. This is illustrated using assays that quantify all transcript variants for each DO (-Tot). Total DO mRNA expression does not realistically reflect relative expression of any given transcript, which is most obvious for Ido2 where Ido2-v1 is highest in the frontal cortex whereas Ido2-FL and Ido2-Tot are highest in the liver.

Given these major differences across tissues and two brain regions, we analyzed additional brain regions for the same DO transcripts. As shown in bottom of Fig. 2, each region of the mouse brain has a distinct pattern of DO expression. All brain regions from naïve mice have essentially no Ido1-FL transcript expression (frontal cortex average *C*_t_ = 38.9, see the figure for the average *C*_t_ values of each transcript in the frontal cortex). All four brain regions express varying levels of the other DO transcripts. The frontal cortex is not particularly abundant for any DO transcript; however, the hypothalamus and striatum are enriched for Ido1-v1 and Ido2-FL expression, whereas the hippocampus and to a much lesser extent the hypothalamus displays enriched Tdo2-FL expression. Tdo2-v1 is similarly expressed across these four brain regions. Ido1-Tot reflects Ido1-v1, because of the absence of Ido1-FL. As such, a qPCR assay for Ido1-v1 or Ido1-Tot would not detect the essential absence of Ido1-FL across these brain regions. Ido2-Tot reflects Ido2-v1 because of the relatively high expression of the variant compared Ido2-FL. Once again, note that assaying brain regions with assays for Ido2-v1 or Ido2-Tot would not detect the difference in relative abundance of Ido2-FL. Tdo2-Tot expression reflects Tdo2-FL because of the similar expression of the variant across brain regions. Therefore, assaying brain regions with Tdo2-FL or Tdo2-Tot assays would not detect the constant expression of Tdo2-v1 across brain regions.

The cause of the distribution differences noted above is unknown, but it is clear that results will vary dependent on the design of the qPCR assay. As a first step to understand the differential regulation of DO expression, qPCR assays designed to quantify the various DO transcripts (Fig. 1 bottom) were used to test for differences in DO regulation in the hippocampus. Given that Ido1 and Ido2 have previously been shown to be induced by inflammatory signals, whereas Tdo2 is purportedly corticosteroid dependent, we next investigated the individual and combined actions of inflammatory mediators and corticosteroids on DO expression using OHSCs.

### Dex and Cort elicit expected anti-inflammatory and negative feedback responses by OSHCs

**Fig. 3:**
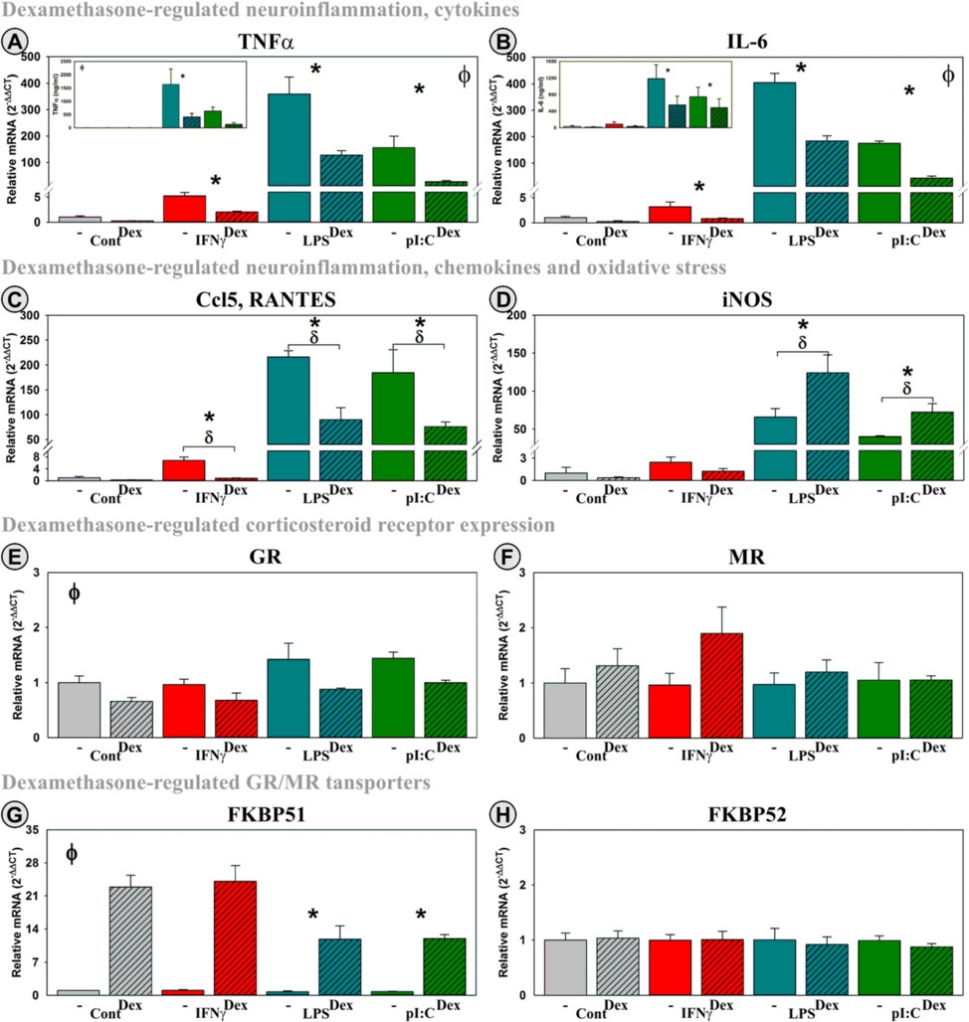
Within OHSCs, Dex elicits a prototypical anti-inflammatory action and acts to down-regulate its own action. OHSCs were treated with three inflammatory mediators (IFNγ, LPS, or pI:C) ± Dex. Gene expression of cytokines (TNFα (**a**) and IL-6 (**b**)), a chemokine (Ccl5 (**c**)), inducible nitric oxide synthase 2 (iNOS, Nos2 (**d**)), glucocorticoid receptor (GR; Nr3c1 (**e**)), mineralocorticoid receptor (MR; Nr3c2 (**f**)), and FK506 binding protein 5 (fkbp5; FKBP51 (**g**)) and 4 (fkbp4; FKBP52 (**h**)) was quantified as were protein levels for TNFα and IL-6 in conditioned media (*inset*). Expression levels are relative to control (no inflammatory mediator and no corticosteroid) samples normalized to 1.0. *p ≤ 0.05 for the main effect of an inflammatory mediator, ^ϕ^ *p* ≤ 0.05 for the main effect of Dex; ^δ^ *p* ≤ 0.05 post hoc analysis for an interaction effect for Dex within inflammatory mediator

We previously characterized the pro-inflammatory responsiveness of OHSCs by demonstrating that TNFα, IL-6, and iNOS expression is induced by both LPS [30] and IFNγ [49]. To confirm responsiveness of our slice cultures to corticosteroids, we first tested for the widely accepted anti-inflammatory role of corticosteroids and the ability of corticosteroids to elicit a negative feedback loop. The glucocorticoid receptor (GR) agonist dexamethasone (Dex), mineralocorticoid receptor (MR) agonist aldosterone (Aldo), or corticosterone (Cort; which binds to both receptors with varying affinity; MR > GR) were tested in the presence or absence of inflammatory mediators (IFNγ, LPS, and pI:C). There was a significant inflammatory mediator main effect on both TNFα and IL-6 mRNA gene expression and protein secretion. IFNγ, LPS, and pI:C induced TNFα, IL-6, and RANTES mRNA expression (Fig. 3a–c, respectively, *p* ≤ 0.01) as well as TNFα and IL-6 protein secretion (insets). Dex significantly (*p* < 0.001) decreased the ability of IFNγ, LPS, and pI:C to induce cytokine/chemokine mRNA (Fig. 3a–c) and protein expression (insets). Corticosterone had essentially the same effect as Dex (not shown). Aldo did not affect cytokine or chemokine expression or secretion (not shown). Interestingly, Dex enhanced the ability of LPS and pI:C to induce iNOS expression although Dex was ineffective when added alone (Fig. 3d), indicating that signaling through the GR can potentiate some inflammatory responses.

OHSCs express both the GR (Nr3c1) and MR (Nr3c2). As expected, GR expression was decreased by Dex (*p* < 0.001), a negative feedback response, but was unaffected by inflammatory mediators (Fig. 3e). Cort mimicked this effect, whereas Aldo was inactive (not shown). MR expression was unaffected by these treatments or their combinations (Fig. 3f). These data indicate that GRs are expressed and active as would be necessary for an anti-inflammatory effect elicited by Dex or Cort, but not Aldo. The lack of an anti-inflammatory effect of Aldo is not due to the absence of the MR expression by OHSCs, but an anti-inflammatory response was not expected by MR activation. FK506 binding protein 5 (fkbp5; FKBP51) and 4 (fkbp4; FKBP52) are considered regulators of corticosteroid receptor activity. FKBP51 acts as negative regulator by preventing translocation of corticosteroid receptor complexes (CR:R) to the nucleus whereas FKBP52 serves as a positive regulator by binding to and translocating the CR:R complex to the nucleus to self-regulate their activity primarily via the regulation of FKBP51 expression [50]. To confirm that this pathway was intact in OHSCs, we quantified FKBP51 and FKBP52 expression. Similar to other reports, we found that Dex (Fig. 3g) increased expression of FKBP51 (*p* < 0.001). The Dex effect was diminished in the presence of LPS and pI:C. Cort, but not Aldo (not shown), mimicked this response. Neither Dex (Fig. 3h), Cort (not shown), Aldo (not shown), nor inflammatory mediators affected the expression of FKBP52.

Together, the data in Fig. 3 establish the ability of corticosteroids to block or enhance specific inflammatory responses (↓ cytokines, ↓ chemokine, and ↑ iNOS). Corticosteroids also diminish (GR) or enhance (FKBP51) expression of genes that are part of a negative feedback regulation of corticosteroid action. Inflammatory mediators can attenuate corticosteroid action (↓ FKBP51). This characterization is necessary to confirm that the OHSC response is similar to those reported in both animal systems and other in vitro models are intact. These confirmatory data were then used to investigate the interaction between corticosteroids and inflammatory mediators on the expression of genes within the *Kynurenine Pathway*.

### Dex, Cort, and Aldo interact with IFNγ to induce Ido1 expression

**Fig. 4:**
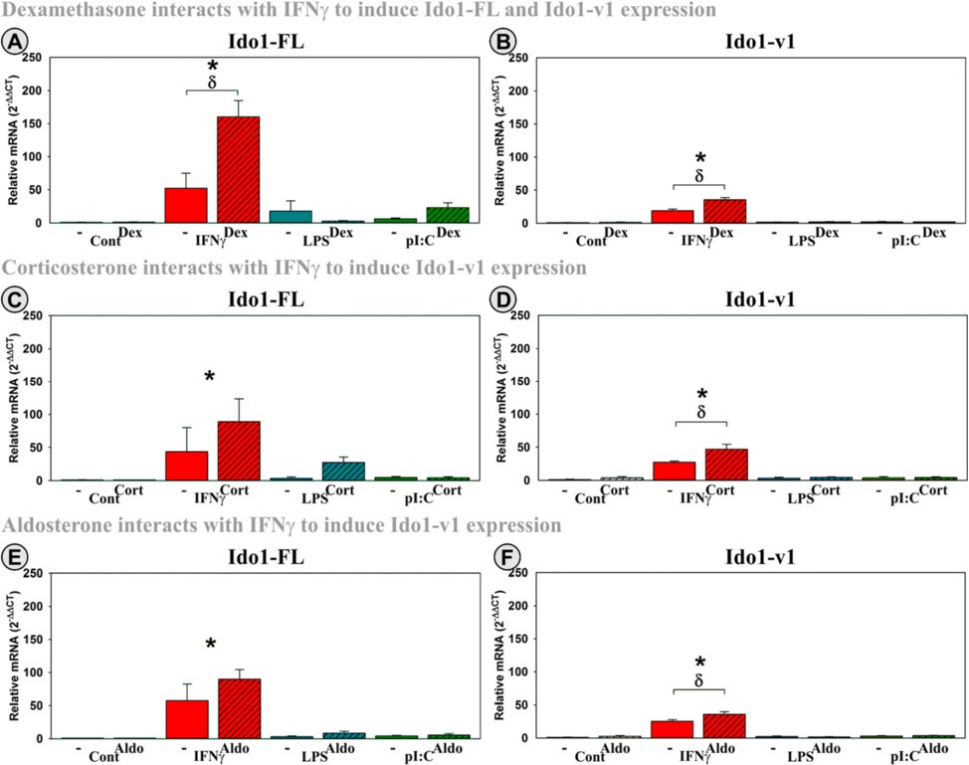
Two distinct Ido1 transcripts are expressed by hippocampal slice cultures, with IFNγ being a strong inducer of Ido1 expression and synergistic with corticosteroids. OHSCs were treated with IFNγ, LPS, and pI:C ± Dex, Cort, or Aldo. Expression levels of two Ido1 transcripts (**a**, **c**, **e**: Ido1-FL; **b**, **d**, **f**: Ido1-v1) are relative to control samples normalized to 1.0. *p ≤ 0.05 for the main effect of an inflammatory mediator, ^ϕ^ *p* ≤ 0.05 for the main effect of either Dex, Cort, or Aldo; ^δ^ *p* ≤ 0.05 post hoc analysis for an interaction effect for Dex, Cort, or Aldo within inflammatory mediator

In control OHSCs, Ido1-FL was essentially absent (*C*_t_ values ≥37), but Ido1-v1 was detectible (*C*_t_ ≤ 34), a finding that closely mimics relative expression found in brains of naïve mice (Fig. 2) validating the use of OHSCs as a model to quantify Ido1 expression. As we have previously shown [49], IFNγ induced Ido1-FL expression (Fig. 4a, c, and e). IFNγ also induced Ido1-v1 (Fig. 4b, d, and f) in OHSCs, although the fold increase was not as large as for Ido1-FL. Alone, neither Dex (A, B), Cort (C, D), nor Aldo (E, F) changed Ido1-FL or Ido1-v1 expression. Interestingly, there was a significant interaction of Dex with inflammatory mediators on Ido1-FL (*F*_(3,22)_ = 8.1, MSE = 6,120, *p* < 0.001) and Ido1-v1 expression (*F*_(3,22)_ = 15.0, MSE = 130.0, *p* < 0.001). By post hoc analysis, Dex significantly accentuated the ability of IFNγ to induce expression of Ido1-FL (*p* < 0.001) and Ido1-v1 (*p* < 0.001). Although Cort and Aldo mimicked this Dex by IFNγ interaction, neither reached significance for Ido1-FL. However, there were significant interactions between Cort (*p* < 0.001) and Aldo (*p* < 0.05) with IFNγ on Ido1-v1 expression. Both steroids significantly accentuated the ability of IFNγ to induce expression of Ido1-v1. LPS and pI:C did not induce Ido1-FL or Ido1-v1 expression, nor did they interact with Cort, Dex, or Aldo. Collectively, these data suggest a unique regulatory pattern for Ido1 expression. Unlike cytokines whose expression is diminished by Dex and Cort, Ido1-FL transcript expression is accentuated by corticosteroids via GR activation, i.e., by Dex and Cort. Unlike iNOS whose expression is increased only by Dex via the GR, Ido1-v1 expression is increased by Dex, Cort, and Aldo, suggesting mediation by the GR, GR/MR, and MR, respectively.

### Inflammatory mediators and Aldo induce Ido2 transcripts

**Fig. 5:**
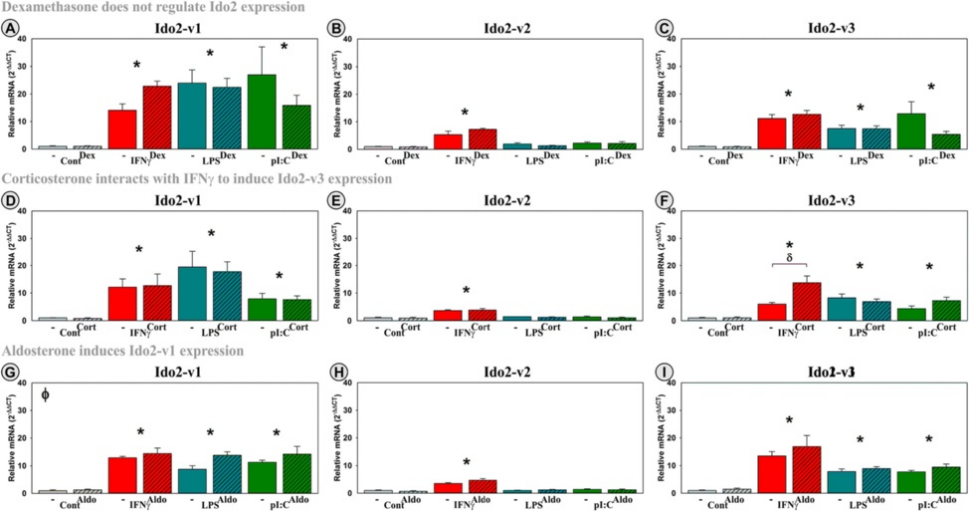
Three distinct Ido2 transcripts are expressed by hippocampal slice cultures, with IFNγ, LPS, pI:C, and Aldo inducing expression of specific Ido2 transcripts. OHSCs were treated with IFNγ, LPS, and pI:C ± Dex, Cort, or Aldo. Expression of Ido2 transcripts (**a**, **d**, **g**: Ido2-v1; **b**, **e**, **h**: Ido2-v2; **c**, **f**, **i**: Ido2-v3) are relative to control samples normalized to 1.0. *p ≤ 0.05 for the main effect of an inflammatory mediator; ^ϕ^ *p* ≤ 0.05 for the main effect of Dex, Cort, or Aldo; ^δ^ *p* ≤ 0.05 post hoc analysis for an interaction effect for Dex, Cort, or Aldo within inflammatory mediator

Ido2-FL was essentially absent (*C*_t_ values ≥37) in OHSCs and was not inducible by inflammatory mediators, corticosteroids, or their combination (not shown). In contrast, Ido2-v1 (*C*_t_~32), Ido2-v2 (*C*_t_~33), and Ido2-v3 (*C*_t_~33) were all detectable in OHSCs. The lower *C*_t_ of Ido2-v1 compared to Ido2-FL of OHSCs agrees with the relative *C*_t_ levels in the hippocampus of naïve mice (Fig. 2) validating the use of OHSCs as a model to quantify Ido2 expression. Ido2-v1 (Fig. 5a, d, and g), Ido2-v2 (Fig. 5b, e, and g), and Ido2-v3 (Fig. 5c, g, and i) expression was induced by IFNγ (*p* < 0.001), with the fold induction of Ido2-v1 (~12-fold) and Ido2-v3 (~10-fold) being greater than that for Ido2-v2 (~threefold). In contrast to Ido1 expression, LPS and pI:C induce Ido2-v1 (*p* < 0.001) and Ido2-v3 (*p* < 0.001) expression, but like Ido1, they do not increase Ido2-v2 expression. Interestingly, Dex did not significantly change Ido2 expression, although there was a significant interaction between Cort and inflammatory mediators for Ido2-v3 expression (*F*_(3,23)_ = 5.6, MSE = 24.4, *p* < 0.01). By post hoc analysis, Cort significantly accentuated the induction of Ido2-v3 in the presence of IFNγ (*p* < 0.001). Cort did not alter LPS nor pI:C action. There was neither a Cort (A, B, C) nor Dex (D, E, F) main effect nor a significant interaction between either Cort or Dex and inflammatory mediators for Ido2-v1 and Ido2-v2. However, there was a small but significant main stimulatory effect of Aldo on Ido2-v1 expression (Fig. 5g) (*p* < 0.05). Thus, although there was not a significant interaction between Aldo and inflammatory mediators, MR activation may enhance Ido2-v1 expression. These data suggest that the regulation of Ido2 expression is distinct from Ido1. Ido2 does not appear to be responsive to GR activation, although MR activation has a mild stimulatory effect that is highly dependent upon the transcript being assessed. Importantly, the three Ido2 transcripts detected in OHSCs are differentially regulated by IFNγ, LPS, and pI:C. This later finding illustrates again that qPCR assay design will affect the response elicited in a treatment-dependent manner.

### Dex and Cort induce Tdo2-FL expression

**Fig. 6:**
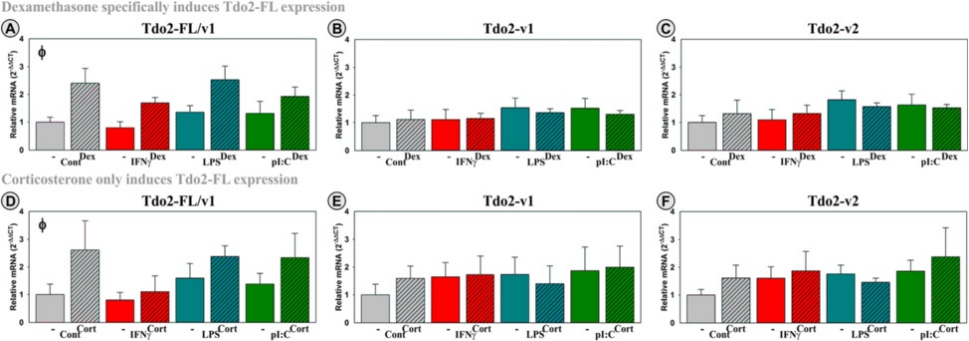
Three distinct Tdo2 transcripts are expressed by hippocampal slice cultures with Cort and Dex inducing only Tdo2-FL. OHSCs were treated with IFNγ, LPS, and pI:C ± Dex or Cort. Expression levels of Tdo2 transcripts (**a**, **d**: Tdo2-FL/v1; **b**, **e**: Tdo2-v1; **c**, **f**: Tdo2-v2) are relative to control samples normalized to 1.0. ^ϕ^ *p* ≤ 0.05 for the main effect of either Dex or Cort

A few studies have suggested that glucocorticoids regulate Tdo2 expression by peripheral tissues [41, 42]; however, corticosteroid regulation of specific Tdo2 transcripts has not been reported. Here, we show that all three Tdo2 transcripts are expressed by OHSCs with varied amplification results; average *C*_t_’s were ~29, ~25, and ~25 for Tdo2-FL/v1, Tdo2-v1, and Tdo2-v2, respectively. Thus, Tdo2-v1 has a lower *C*_t_ value than Tdo2-FL/v1 in both OHSCs and in the hippocampus of naïve mice (Fig. 2), again validating the use of OHSCs as a model to quantify DO expression. Addition of IFNγ, LPS, and pI:C did not alter the expression of any Tdo2 transcripts (Fig. 6a–f), but both Dex (Fig. 6a; *p* < 0.001) and Cort (Fig. 6d; *p* ≤ 0.05) induced Tdo2-FL/v1 expression. As Tdo2-FL/v1 expression is shown to be highest in the hippocampus (Fig. 2), a brain region with a high density of both GRs and MRs [51, 52], it is not surprising that both Dex and Cort induce Tdo2-FL. Aldo did not alter Tdo2-FL expression (not shown), indicating that the stimulatory effects of Cort and Dex are mediated by the GR. Surprisingly, Tdo2-v1 and Tdo2-v2 expression were not regulated by corticosteroids. Since the assay for Tdo2-FL also amplifies Tdo2-v1, the latter having a lower *C*_t_ and thus possible greater expression level, the true inductive power of Cort and Dex on Tdo2-FL expression is diluted by the concurrent detection of the non-inducible Tdo2-v1 transcript. This finding confirms that the Tdo2-FL sequence is not just a truncated/incomplete version of Tdo2-v1, but that Tdo2-FL is a unique transcript under distinct regulatory control compared to its two counterparts. These results also indicate that quantifying corticosteroid induction of Tdo2 is only feasible when assessing expression of the Tdo2-FL transcript.

### Changes in the genetic expression of enzymes downstream of DOs along the *Kynurenine Pathway*

**Fig. 7:**
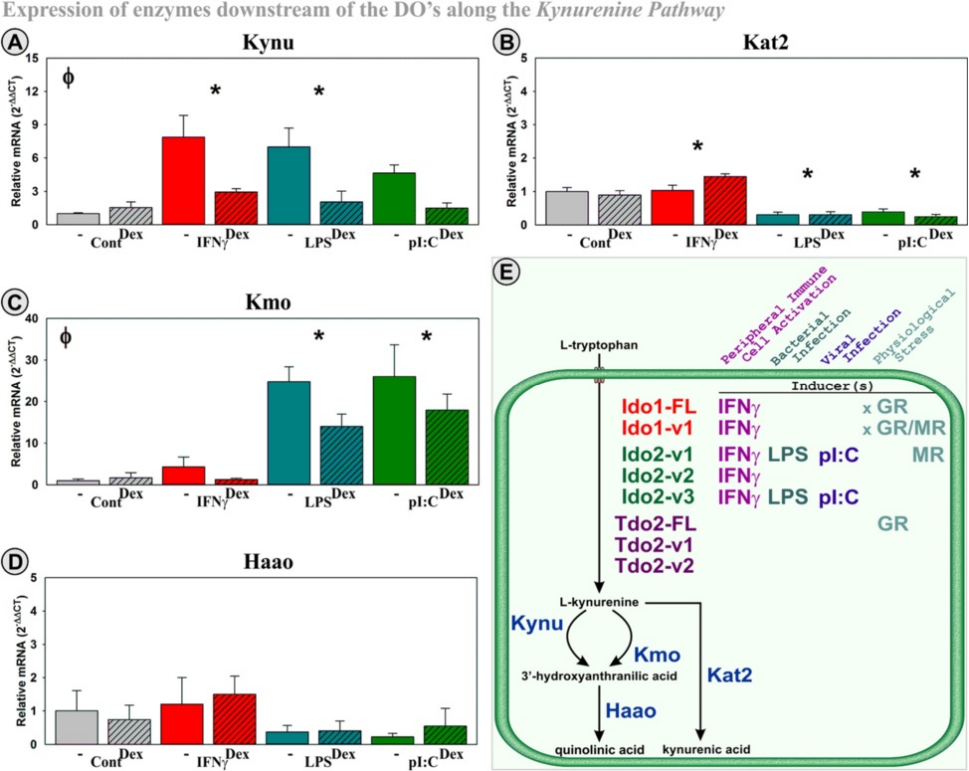
Regulation of mRNA expression of enzymes downstream of the DOs in the *Kynurenine Pathway* in hippocampal slice culture. OHSCs were treated with IFNγ, LPS, and pI:C ± Dex. Kynu (**a**), Kat2 (**b**), KMO (**c**) and Haao (**d**) expression was quantified relative to control samples normalized to 1.0. *p ≤ 0.05 for the main effect of an inflammatory mediator, ^ϕ^ *p* ≤ 0.05 for the main effect of Dex

There are several enzymes involved in the metabolism of tryptophan along the *Kynurenine Pathway*. Although the DOs are rate limiting for entry of tryptophan into the pathway, downstream enzymes are necessary for the conversion of Kyn to either QuinA or KynA. Kat2, Kynu, KMO, and Haao are involved in the conversion of Kyn neuroactive metabolites (Fig. 7 picture insert). By analyzing the expression pattern of three of these enzymes, we further emphasize the unique transcriptional regulation of the DOs. None of the downstream enzymes were inducible by Dex, Cort, or Aldo, and none showed the positive interaction between inflammatory mediators and GR or MR activation. There was an inflammatory main effect on KMO and Kynu expression in which both LPS and pI:C significantly induced the expression of KMO (Fig. 7a) (*p* < 0.01) while IFNγ and LPS significantly increased Kynu (Fig. 7b) (*p* < 0.05). Dex decreased the expression of KMO (*p* < 0.05) and Kynu (*p* < 0.001). Haao expression was not changed by any of the treatments nor their combination (Fig. 7c). In contrast, LPS and pI:C significantly decreased Kat2 expression (*p* < 0.001), without an effect of Dex (Fig. 7d). The actions of Dex on Kynu and KMO expression were mimicked by Cort, but not Aldo (not shown). These findings support the hypothesis that corticosteroids regulate expression of KMO and Kynu enzymes via the GR. Overall, the stimulatory effect of inflammatory mediators on Kynu and KMO expression in parallel with a decrease in Kat2 expression should shift Kyn metabolism towards QuinA production (Fig. 7 insert). This effect would be somewhat tempered by the inhibitory effect of Dex on Kynu and KMO expression. More importantly, the non-rate limiting enzymes are not susceptible to the positive interaction between IFNγ and corticosteroids to induce Ido1 or the induction by Aldo or Dex seen with Ido2 and Tdo2, respectively.

## Discussion

Induction of the *Kynurenine Pathway* by inflammatory stimuli or stress has been associated with the development of depression-like behaviors [26–30, 33]. An understanding of the regulation of enzymes within this pathway will aid in discovering the mechanism for the etiology of inflammation-induced depression. This is the first study to (1) design assays for specific mRNA transcript variants of Ido1, Ido2, and Tdo2, (2) describe tissue specificity for each variant, and (3) uncover a novel interaction between inflammatory mediators and stress hormones to induce specific Ido1, Ido2, and Tdo2 mRNA transcript variants. OHSCs were used to model how the hippocampus responds to corticosteroids and inflammatory stimuli. We validated the OHSC model by showing that GR, but not MR, activation elicits an anti-inflammatory response while initiating a negative feedback loop (↓ GR, ↑ FKBP51). Thus, OHSCs maintain corticosteroid responses that occur in the intact murine brain. Unexpectedly, LPS- and pI:C-induced iNOS expression was accentuated by Dex. Microglial iNOS expression is induced by LPS but attenuated by Dex [53, 54]. However, after 6 h (as in the current study), Dex did not attenuate the LPS-induced iNOS promoter activity in astrocytes [55]. Thus, not all cells respond with a diminution of iNOS expression. The cell type, or cell-cell interaction within OHSCs, that mediates Dex-enhanced iNOS expression is unknown at this time.

In contrast to the expected anti-inflammatory response, Dex, Cort, and Aldo amplified the ability of IFNγ to increase Ido1 expression, establishing that both GR and MR activation are involved in the induction of Ido1. Whether corticosteroids synergize with IFNγ to induce Ido1-FL or Ido1-v1 expression by a specific cell type(s) is unknown. Additionally, we found that OHSCs express three Ido2 transcripts. IFNγ-induced expression of all three transcripts whereas LPS and pI:C increased expression of only two transcripts. Aldo and Cort, but not Dex, upregulated the expression of specific Ido2 transcripts although these responses were muted compared to that of Ido1. Aldo moderately induced expression of Ido2-v1 independent of inflammation, while Cort interacted with IFNγ to accentuate expression of Ido2-v3. These findings suggest a small but significant MR-mediated regulation of Ido2. Finally, OHSCs express three Tdo2 transcripts; Cort and Dex (not Aldo) increase expression of only one Tdo2 transcript, indicating a GR-mediated stimulatory response. These important data are essential in further understanding the regulation of the *Kynurenine Pathway* in the brain and detailing the importance of investigating post-transcriptional regulation of the DO genes.

Ido1 expression is induced by IFNγ in human and mouse microglia, macrophages, astrocytes [56–60], neurons [61], brain endothelial cells [62], and mouse OHSCs ([45]; Fig. 4). However, although extremely critical to our understanding of DO regulation, none of these manuscripts provide enough information to determine basal expression or to identify variations in transcript(s) responsiveness to IFNγ. In the current work and as suggested in the literature, Ido1-FL mRNA expression is extremely low to undetectable in the naïve mouse brain (Fig. 2) [63, 64], including the hippocampus [65]. Although there is essentially no Ido1-FL mRNA in the naïve mouse brain, there is Ido1 enzymatic activity [66–68]. Enzymatic activity attributed to Ido1 in the naïve must derive from the protein product of Ido1-v1. Similarly, we have shown that control OHSCs have Ido1 enzymatic activity [30]; OHSCs express Ido1-v1 but not Ido1-FL (Fig. 3). As such, basal Ido1 activity within OHSCs (like naïve mouse brain) must be mediated by the Ido1-v1-encoded protein. Confirmation of this conclusion awaits overexpression of this variant and assessment of its activity. Austin [44] overexpressed a ~51 kDa Ido1-FL protein, whereas Ball [24] and Pallotta [45] overexpressed ~45 kDa proteins. Whether the enzymatic activity of the smaller proteins was due to Ido1-FL- or Ido1-v1-derived product or whether the two products have similar specific activities was unclear.

We find Ido1-v1 in all brain regions examined (Fig. 2) with particular enrichment in the striatum and hypothalamus. The relevance of this finding relative to animal behavior, especially relative to inflammation- and stress-induced depression-like behavior and innate immune responses in the CNS, warrants further investigation. To determine the human relevance of DO transcript regulation, we have generated assays to measure human Ido1, Ido2, and Tdo2 variant transcripts. We detected multiple Ido1, Ido2, and Tdo2 transcripts (unpublished data) in human cells. Several Ido1 and Ido2 transcripts were IFNγ inducible and further induced by corticosteroids. This work suggests that the complex regulation of DO transcripts that we have identified in the mouse hippocampus is also present in human cells. Relevance of these finding to psychiatric diseases warrants further investigation.

Glucocorticoid response elements (GRE) modulate GR and MR binding to promoters to directly affect gene transcription. Using Motif Map, we found no evidence for a GRE within the mouse Ido1 or Ido2 genes. This finding suggests that corticosteroid:receptor complexes (C:GR or C:MR) are not binding directly to the Ido1 or Ido2 promoter but are more likely to interact with the IFNγ signaling cascade to accentuate Ido1 and Ido2 expression. IFNγ activates the Janus kinase/signal transducer and activator of transcription (JAK/STAT) pathway, and C:GR’s can enhance JAK/STAT signaling to increase gene transcription independent of GREs [69–73]. These published mechanisms suggest that GR activation (by Dex or Cort) enhance Ido1-FL, Ido1-v1, Ido2-v3, and iNOS expression via JAK/STAT. To date, the C:MR complex has not been shown to directly modulate the JAK/STAT pathway although we did find that Aldo interacts with IFNγ to induce Ido1-v1 expression. The mechanism by which Aldo and IFNγ interact to induce Ido1-v1 or Aldo acts independently to induce Ido2-v1 expression is unclear at this time. Independent of the mechanism, our findings are important because they illustrate a multifaceted interaction between corticosteroids and IFNγ to upregulate genes along the *Kynurenine Pathway*, particularly the rate-limiting enzymes Ido1 and Ido2.

Ido2-FL mRNA has been reported as low to undetectable in the naïve mouse brain [24, 64]. However, in a separate study using a probe that detects all Ido2 transcripts, Ido2 mRNA was detected in neurons of the cerebral cortex and cerebellum [74]. Using qPCR, we find Ido2-FL primarily in the mouse striatum and hypothalamus, whereas Ido2-v1 is expressed uniformly across at least four brain regions (Fig. 2). Only variant Ido2 transcripts were detectable in OHSCs. Unlike Ido1, Ido2 transcripts are induced by LPS and pI:C, in addition to IFNγ. Also, Ido2-v1 expression is increased by Aldo alone. However, similar to Ido1, Ido2-v2 was induced only by IFNγ. Thus, it is clear that Ido2 is regulated in a transcript-specific manner by both inflammatory mediators and corticosteroids.

Proteins encoded by Ido2-FL and Ido2-v3 are enzymatically active although the Ido2-FL product has higher activity/unit protein than the variant protein encoded by Ido2-v3 [47]. This result suggests that differential expression of Ido2 transcripts will have profound effects on net enzymatic activity. Thus, it is critical to evaluate expression of all transcripts and ultimately the enzymatic activity of each protein variant. Clearly an assay such as the one that quantifies all three Ido2 transcripts (Ido2-Tot) does not accurately reflect changes in expression of any given variant (Fig. 2), nor would it be expected to parallel enzymatic activity.

The presence of a significant LPS and pI:C effect on Ido2 expression may relate to an undiscovered role for Ido2 during bacterial and viral infections within the brain. The induction of Ido1 and Ido2 transcripts by IFNγ within OHSCs mimics the cytokine-mediated response of the brain to a peripheral infection. This induction is critical to the development of depression-like behaviors [28, 35]. However, based upon data in this report, it is quite possible that specific Ido2 transcripts will be induced by bacterial (e.g., LPS) or viral (e.g., pI:C) infection within the brain. To date, there are no studies describing behavioral responses associated with the induction of Ido2, due to lack of Ido2-specific inhibitors and only the recent development of Ido2^KO^ mice [47]. Thus, the role of Ido2 in inflammation-dependent psychiatric diseases is unknown.

Tdo2 mRNA is present throughout the rodent brain [48, 75]. Similar to the study by Kanai [48], our results suggest that there are unique and unappreciated roles for the various Tdo2 transcripts within the brain. Tdo2-FL and Tdo2-v1 encode the same mature protein, while Tdo2-v2 encodes an N-terminal truncated “variant” protein. Both Tdo2 protein isoforms have similar enzymatic activity [48]. Since any qPCR assay designed to quantify Tdo2-FL also assays Tdo2-v1, it is impossible to independently assess Tdo2-FL expression. However, Tdo2-FL is not a simple incomplete sequence of Tdo2-v1 but is rather a unique transcript that is controlled by specific regulatory mechanisms. As supported by our OHSCs data, the expression of only Tdo2-FL is induced by Dex and Cort. This inductive response is probably a direct effect of Dex and Cort on GR activation as a GRE motif has been predicted within the Tdo2 gene [76, 77].

There is a significant amount of research suggesting that elevated Ido1 and Ido2 is associated depression-like behavior [28, 35] and elevated Tdo2 expression is linked to Schizophrenia [78, 79]. With very minimal information on the activity of the proteins translated from variant DO transcripts, the results presented in this manuscript are only the first step of many necessary to define their role in depression or other psychiatric diseases. Although it is generally believed that DO enzymatic activity within the brain of naïve and experimental mice [66–68] results from the protein product of Ido1-FL, our data suggest that the expression of other DO variants may be responsible for Kyn production within the brain. Ascertaining the enzymatic activity of each transcript product is outside the scope of this study, but such knowledge would greatly add to the understanding of the functional relevance of the DOs and their relevance to psychiatric diseases.

## Conclusions

In conclusion, our data support a role of the *Kynurenine Pathway* in major depression [80] and suggest that inflammatory and corticosteroid pathways converge to induce DO expression. This convergence has not been previously tested. Our data show that Ido1, Ido2, and Tdo2 are differentially regulated (summarized in Fig. 7e). Ido1 induction by IFNγ is enhanced by GR and MR activation, suggesting that stress hormones interact with IFNγ to enhance kynurenine synthesis within the hippocampus. In contrast, Ido2 transcripts are activated by IFNγ, LPS, and pI:C. Thus, Ido2 expression is responsive to peripheral immune-cell-derived IFNγ (resident cells in the brain do not produce IFNγ) and bacterial or viral infections (via LPS or dsRNA) but relatively unaffected by stress hormones. Finally, Tdo2 transcripts are either unchanged (v1 and v2) or induced by GR activation (Tdo2-FL) indicating a unique stress responsiveness. Associating physiological and behavioral consequences to the differential regulation of DO transcripts is beyond the scope of this manuscript. However, these data show that rate-limiting enzymes for tryptophan metabolism along the *Kynurenine Pathway* can be differentially regulated to allow the brain to specifically respond to various challenges. Quantifying the expression of a single DO or a single DO transcript will not accurately reflect the true expression of the tryptophan-degrading enzymes. Thus, it is imperative to evaluate the expression of each transcript for both animal and in vitro studies. The current study validated assays that are needed to achieve this goal. It is also the first work to describe Ido1, Ido2, and Tdo2 variant transcripts within the brain.

## Declarations

This work was supported by RO1 MH083767 and RO1 MH101145 to RHM and R01 SUB UT 00000712 to KWK.
